# PPARγ Deficiency in Carbon Nanotube-elicited Granulomatous Inflammation Promotes a Th17 Response to a Microbial Antigen

**DOI:** 10.35248/2157-7439.20.11.541

**Published:** 2020-02-18

**Authors:** Victoria Sanderford, Barbara P Barna, Robert A Barrington, Anagha Malur, Arjun Mohan, Nancy Leffler, Eman Soliman, Mary Jane Thomassen

**Affiliations:** 1 Program in Lung Cell Biology and Translational Research, Division of Pulmonary, Critical Care and Sleep Medicine, Department of Medicine, Brody School of Medicine, East Carolina University, Greenville, NC, USA; 2 Department of Microbiology and Immunology, University of South Alabama, Mobile, AL, USA; 3 Department of Pharmacology and Toxicology, Faculty of Pharmacy, Zagazig University, Zagazig, Egypt

**Keywords:** Multiwall carbon nanotubes, Alveolar macrophages, Lymph nodes, Granulomas, PPAR gamma, Th 17, Sarcoidosis

## Abstract

**Background:**

The pathological consequences of interaction between environmental carbon pollutants and microbial antigens have not been fully explored. We developed a murine model of multi-wall carbon nanotube (MWCNT)-elicited granulomatous disease which bears a striking resemblance to sarcoidosis, a human granulomatous disease. Because of reports describing lymphocyte reactivity to mycobacterial antigens in sarcoidosis patients, we hypothesized that addition of mycobacterial antigen (ESAT-6) to MWCNT might elicit activation in T cells.

**Methods:**

Macrophage-specific peroxisome-proliferator-activated receptor gamma (PPARγ) knock out (KO) mice were studied along with wild-type mice because our previous report indicated PPARγ deficiency in sarcoidosis alveolar macrophages. MWCNT+ESAT-6 were instilled into mice. Controls received vehicle (surfactant-PBS) or ESAT-6 and were evaluated 60 days post-instillation. As noted in our recent publication, lung tissues from PPARγ KO mice instilled with MWCNT+ESAT-6 yielded more intensive pathophysiology, with elevated fibrosis

**Results:**

Inspection of mediastinal lymph nodes (MLN) revealed no granulomas but deposition of MWCNT. MLN cell counts were higher in PPARγ KO than in wild-type instilled with MWCNT+ESAT-6. Moreover, the CD4:CD8 T cell ratio, a major clinical metric for human disease, was increased in PPARγ KO mice. Bronchoalveolar lavage (BAL) cells from PPARγ KO mice instilled with MWCNT+ESAT-6 displayed increased Th17 cell markers (RORγt, IL-17A, CCR6) which associate with elevated fibrosis.

**Conclusion:**

These findings suggest that PPARγ deficiency in macrophages may promote ESAT-6-associated T cell activation in the lung, and that the MWCNT+ESAT-6 model may offer new insights into pathways of lymphocyte-mediated sarcoidosis histopathology.

## INTRODUCTION

Determining the potentially adverse effects of environmental carbon pollutants on human health is a difficult and continuing problem. Production and usage of engineered nanomaterials represent a growing technology [1] while combustion-generated carbon pollution is ubiquitous and can include both carbon nanotubes and nanoparticles [2]. Animal model studies suggest that multiwall carbon nanotubes (MWCNT) can affect immunological responses, produce granulomas, or elicit fibrosis, depending upon chemical and structural characteristics [3,4].

Our laboratory has focused on constructing a murine model of MWCNT-induced chronic pulmonary granulomatous pathology which exhibits many characteristics found in sarcoidosis, a human granulomatous disease of unknown etiology. Epidemiological studies have linked sarcoidosis to conditions in which carbon pollutants may be produced [5–7]. Moreover, carbon nanotubes have been detected in tissues from sarcoidosis cases associated with the 2001 World Trade Center disaster [8].

The findings linking sarcoidosis to environmental carbon pollutants are recent. Sarcoidosis has been recognized since the early 1900s and postulated to be due to infectious agents such as *mycobacterium tuberculosis* (reviewed by Brownell et al) [9]. Further studies have assessed the evidence for mycobacteria in sarcoidosis and have found support for an association between mycobacteria and sarcoidosis pathology [10]. One line of evidence is the presence of adaptive immune responses to mycobacterial proteins in many sarcoidosis patients [11,12]. Rather than intact organisms however, current findings suggest that inert mycobacterial antigens may be the key elements involved in sarcoidosis immunopathology [10].

Pathology associated with sarcoidosis is thought to derive from activation of T cells that potentiate progressive macrophage activation. Subsets of effector T cells are distinguished by particular combinations of transcription factors, cytokines and chemokine receptor profiles associated with each. For example, T helper type 1 (Th1) cells express the transcription factor T-Bet and produce interferon gamma (IFNγ) [13]. A second subset of effector T cell, T helper type 17 (Th17) cells, express transcription factor RORγt, chemokine receptor CCR6, and produce interleukin 17, (IL-17) a cytokine associated with development of fibrosis [14,15]. Importantly, T helper type 1 and 17 cells are observed in bronchoalveolar lavage from sarcoidosis patients, thus implicating them in disease [14]. Recent sarcoidosis studies also implicate Th17.1 cells, a novel T cell subset expressing CCR6 but producing IFNγ [16].

Because of the above findings implicating both carbon pollutants and mycobacterial antigens in sarcoidosis, we examined the effects of adding the mycobacterial peptide, ESAT-6, to MWCNT instillation in wild type C57Bl/6 and in macrophage-specific PPARγ mice. Cells from BAL and mediastinal lymph nodes were analyzed to determine whether a particular T cell subset might be responsible for mediating observed pathology. Both wild-type and PPARγ KO mice display granulomatous lung pathology in response to oropharyngeally instilled MWCNT but exaggerated responses are noted in the PPARγ KO strain [17,18] In addition, our most recent findings also demonstrate elevated fibrosis along with exacerbated granulomatous pathology in MWCNT+ESAT-6 instilled PPARγ KO mice [19]. Because PPARγ deficiency is reported in sarcoidosis [20], we hypothesized that combined instillation of ESAT-6 and MWCNT might elicit immunological changes in macrophage-specific PPARγ-deficient (KO) mice that could resemble those reported in sarcoidosis [11].

## MATERIALS AND METHODS

### MWCNT model

All studies were conducted in conformity with Public Health Service (PHS) Policy on humane care and use of laboratory animals and were approved by the institutional animal care committee. C57BL/6J wild-type from Jackson Laboratories, (Bar Harbor, ME), and macrophage-specific PPARγ KO mice as previously described [21] were utilized in experiments. All studies were conducted using littermates that were sex-matched and/or multiple litters that were age-matched. Mice received an oropharyngeal instillation of MWCNT after sedation with isofluorane. MWCNTs (catalogue number 900–1501, lot GS1802, SES Research, Houston, TX) were freshly prepared and have been extensively described previously [17,19]. A single pulmonary instillation of MWCNT (100 μg) in PBS/35%surfactant (vehicle)+ESAT-6 peptide 14 [NNALQNLARTISEAG] (20 μg) was delivered to wild-type C57Bl/6 mice and PPARγ KO mice as previously described [22]. Sham controls received vehicle alone; additional controls received only ESAT-6 [22]. Animals were sacrificed at 60 days post instillation for collection of bronchoalveolar lavage (BAL) as well as lung and lymph node tissues [22].

### Analyses of lungs and lymph nodes

Leukocyte differential counts of BAL cells were calculated from cytospins; BAL cells were then processed for RT-PCR analysis using the ABI Step One Plus Real Time PCR system (Applied Biosystems, Foster City, CA) as previously described [23]. Primer-probe sets for the following T helper cell markers: T-Bet [PPM03727A], T helper cell type 1 (Th1); interferon-gamma (IFN-γ) [PPM03121A], Th1; signal transducer and activator of transcription (STAT4) [PPM04644B], Th1; Interleukin 4 (IL-4) [PPM03013F], T-helper cell type 2 (Th2); GATA3 [PPM05199A], Th2; RAR-related orphan receptor gamma (RORγt) [PPM25095A], T helper cell type 17 (Th17); IL-17A [PPM03023A], Th17; CCR-6 [PPM03185B], Th17; chemokine ligand 20 (CCL20) [PPM03142B] and interleukin 6 (IL6) [PPM03015A], Th17 development and recruitment; and housekeeping gene GAPDH [PPM02946E] were obtained from Qiagen, Germantown, MD. Data were expressed as fold change in mRNA expression compared to baseline C57Bl/6 PBS/surfactant control values.

Lungs were dissected and fixed in PBS 10% formalin; granuloma scoring was performed on representative Hematoxylin and Eosin (H&E) stained sections as previously described [22]. Prior to lymph node analyses, volume calculations of mediastinal lymph nodes were carried out using the following formula: V= (lxw^2^X**π/6**) (19). Lymph nodes were dissected and fixed overnight in PBS-buffered 10% formalin before embedding in paraffin. Representative histological images were taken for each condition and mouse strain using a Zeiss Axio Imager A1. Lymph nodes were also processed for mass spectrometry as previously described for the lungs [19].

### Characterization of lymph node cells

From each treatment group, single cell suspensions of mediastinal lymph node mononuclear cells (MNC) were isolated by density gradient centrifugation using Lympholyte M (Cedarlane Laboratories, Burlington, NC). Aliquots of node cells were evaluated by RT-PCR for expression of transcription factors and cytokines listed above for BAL cell analysis. For flow cytometric characterization of cell membranes, cells were stained with antiCD4 (clone RM4–5), and anti-CD8 (Clone 53–6.7) antibodies, (eBiosciences, San Diego, CA). Subsequently, cells were analyzed by FACSCanto II and sorted using the multi-laser FACSAria II-SORP housed in the Flow Cytometry Laboratory of the University of South Alabama College of Medicine. Data were analyzed with FlowJo software (Tree Star Ashland, OR).

### Statistical analyses

Data were analyzed by student’s t-test, one-way analysis of variance (ANOVA) (two groups) or two-way ANOVA (> two groups) together with Tukey’s test using Prism 7 software (GraphPad, Inc., San Diego, CA.).

## RESULTS

### Mediastinal lymphadenopathy occurs in wild-type and PPARγ KO mice 60 days after instillation with MWCNT+ESAT-6

We previously reported that instillation of MWCNT with or without ESAT-6 induced pulmonary granuloma formation in both wild-type C57Bl/6 and PPARγ KO mice 60 days later (19;20), though granuloma scores associated with MWCNT+ESAT-6 instillation were significantly (p≤0.05) greater in PPARγ KO mice (5.5 ± 0.2; n=6) than in wild-type (3.5 ± 0.62; n=6). No granulomas appeared in animals instilled with vehicle or ESAT-6 alone as previously reported (19;20).

**Figure 1:**
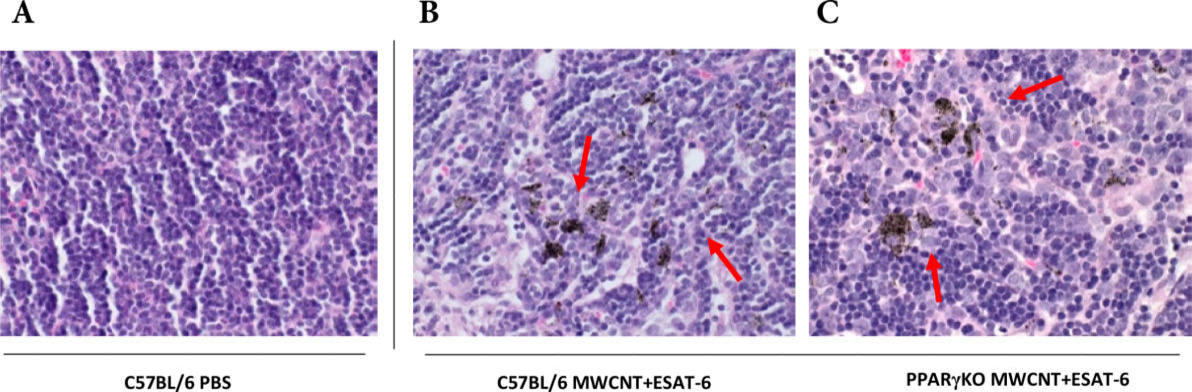
MWCNT are present in mediastinal lymph nodes. Mediastinal lymph node (MLN) histology was examined after Hemotoxylin and Eosin staining of MLN from C57BL/6 wild-type or PPARγ KO mice instilled 60 days previously with PBS/surfactant vehicle (1A) or MWCNT + ESAT-6 (1B, 1C). Carbon particles (MWCNT) are visible within node tissues (arrows) of MWCNT + ESAT-6-instilled mice (1B 1C).

**Figure 2:**
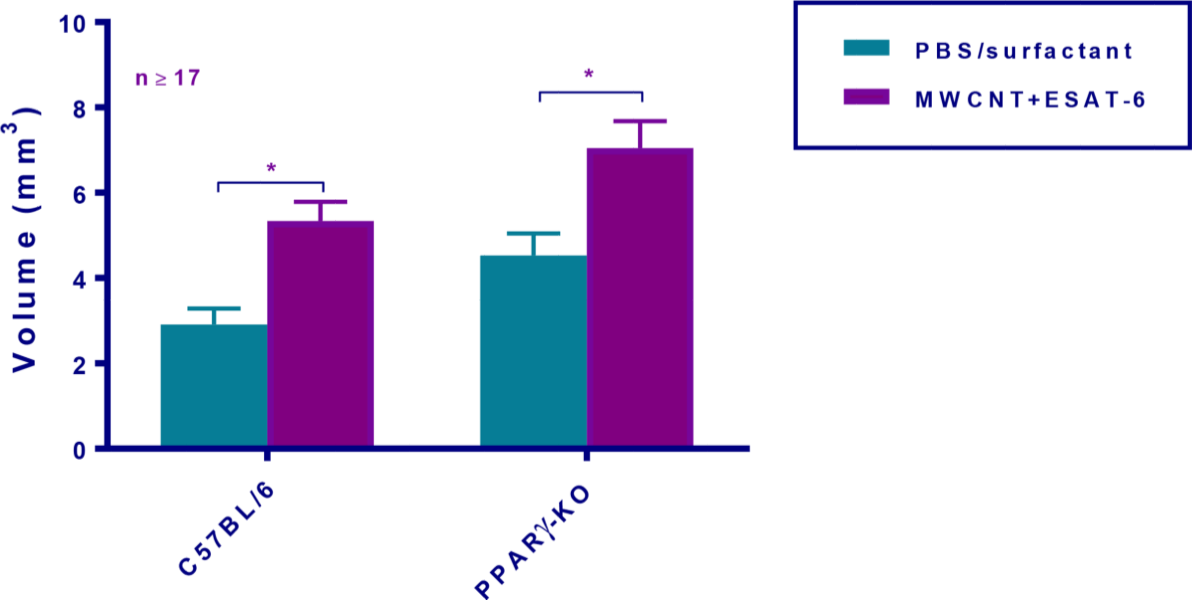
PPARγ deficiency promotes exacerbated lymphadenopathy. Volumetric comparisons of mediastinal lymph nodes (MLNs) were calculated in C57BL/6 and PPARγ KO mice at 60 days post-instillation. Data are represented by means ± SEM.; * indicates p ≤ 0.05.

**Figure 3:**
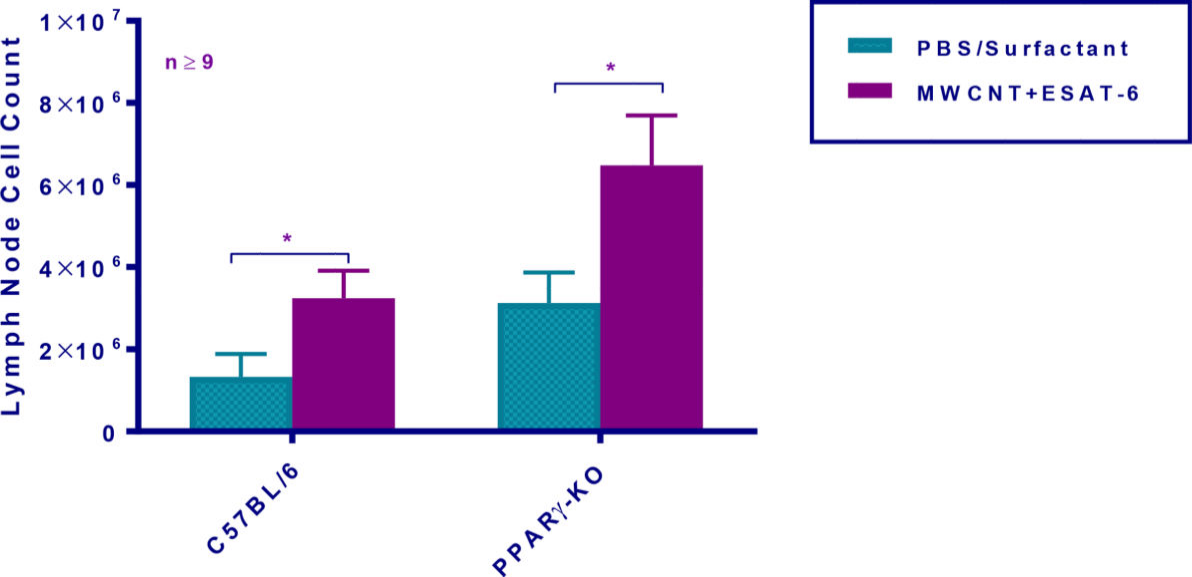
Mediastinal lymph node (MLN) cell counts are increased in PPARγ KO mice. Quantitative comparison of MLN total cell counts was performed in C57BL/6 and PPARγ KO mice at 60 days after instillation. Data are represented by means ± SEM.; * indicates p 0.05.

To determine whether MWCNT were visible in mediastinal lymph nodes (MLN) from mice instilled 60 days previously with MWCNT+ESAT-6, H&E staining and analyses were performed. MWCNT were visible by light microscopy in both wild-type and PPARγ KO mice (Figure 1A–1C). Although MWCNT were detectable, lymph nodes contained no evidence of granulomas or ESAT-6 (as analyzed by mass spectroscopy) (19). Gross examination of MLN indicated lymphadenopathy in both wild-type and PPARγ KO mice instilled with MWCNT+ESAT-6 compared to vehicle control (Figure 2). Lymphadenopathy was not present in mesenteric (gut-associated) lymph nodes from mice (data not shown). Inspection of total cell counts in MLN revealed significantly (p≤ 0.05) elevated values in PPARγ KO mice treated with MWCNT+ESAT-6 compared to comparably treated wild-type mice or to PPARγ KO instilled with vehicle alone (Figure 3). As previously shown [19], ESAT-6 alone was not different from the vehicle control. Wild-type MLN cell counts were also significantly increased by MWCNT+ESAT-6 instillation compared to vehicle alone (Figure 3).

### CD4+ Helper T lymphocytes predominate in PPARγ KO mediastinal lymph nodes

**Figure 4:**
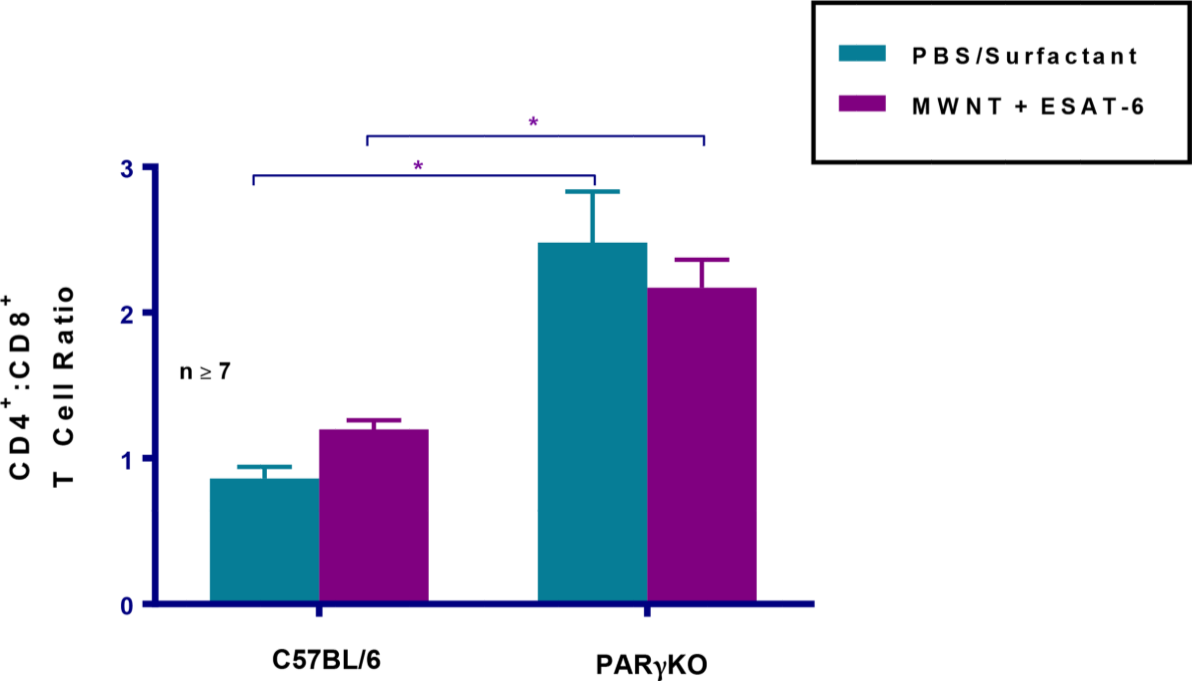
PPARγ KO mice exhibit elevated CD4/CD8 T cell ratios. Quantitative comparisons of CD4/CD8 cell ratios were determined in wild-type and PPARγ KO mice at 60 days after instillation. Data are represented by means ± SEM.; * indicates p ≤ 0.05.

Because an increased ratio of CD4 to CD8 cells is a hallmark of human disease (23), we next determined this ratio in the MWCN-Tinduced mouse model. Analyses of CD4 and CD8 lymphocytes in MLN cells indicated consistent and significant (p ≤0.05) increases in PPARγ KO CD4/CD8 ratios compared to wild-type mice (Figure 4). Thus, increased pathology in macrophage-specific PPARγ KO mice associates with an increased CD4/CD8 ratio.

### BAL cells from MWCNT+ESAT-6 instilled mice express elevated T Helper (Th) Cell transcription factors compared to sham controls

**Figure 5:**
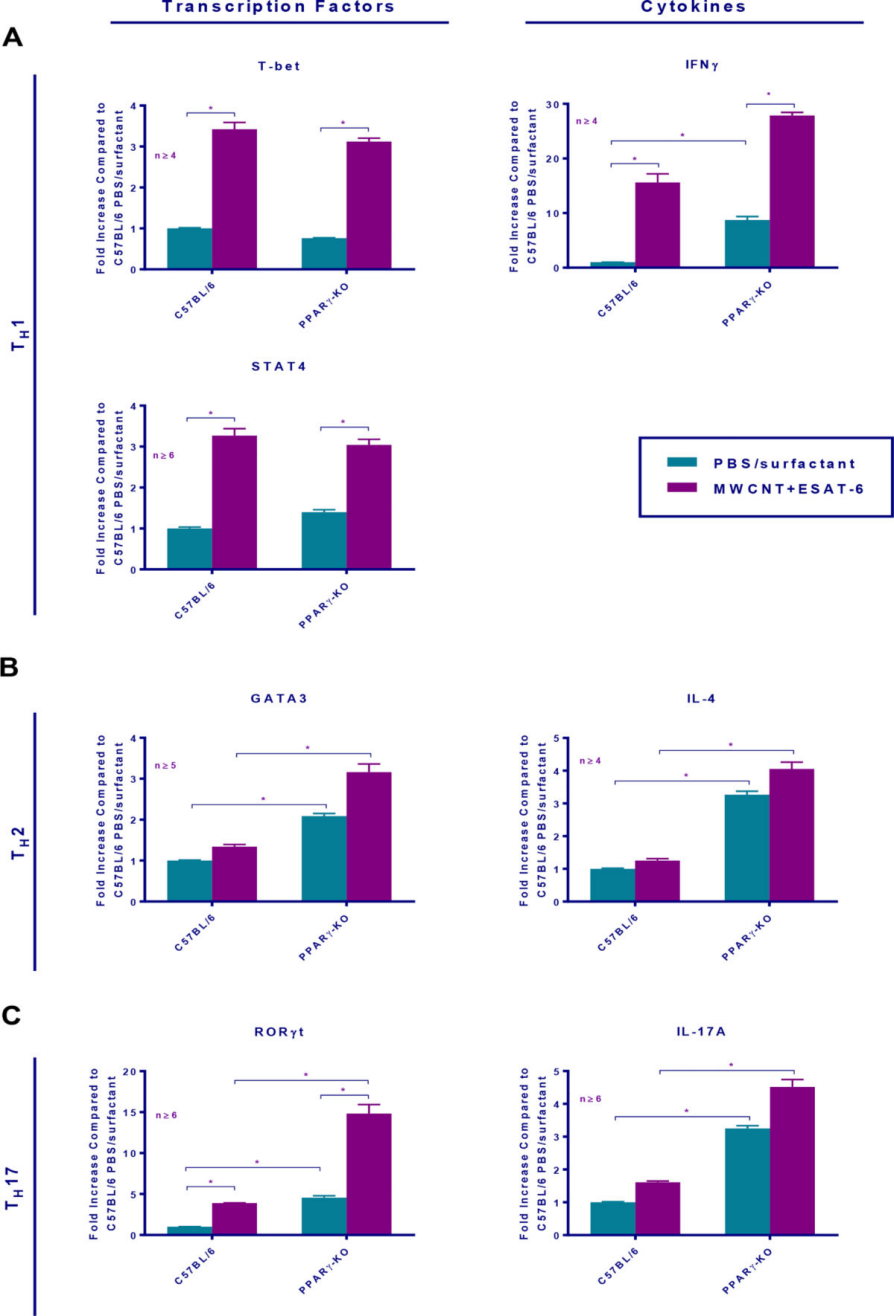
MWCNT + ESAT-6 instillation elevates T cell-associated gene expression in BAL cells from C57Bl/6 and PPARγ KO mice. qPCR analysis of T cell associated transcription factors and cytokines in BAL cells from C57BL/6 and PPARγ-KO mice 60 days post-instillation with PBS/surfactant or MWCNT+ESAT-6. Th1 (5A), Th2 (5B), Th17 (5C). Data are represented by mean relative fold change ± SEM.; * indicates p 0.05.

Our previous reports consistently showed increased T (CD3^+^) cells in lungs of both wild-type and PPARγ KO mice instilled with MWCNT+ESAT-6 compared to lungs from other instilled groups (19;22). Here we hypothesized that lung tissues exposed to MWCNT+ESAT-6 might also express T helper cell factors compared to vehicle (PBS/Surfactant) controls. As noted previously, total BAL cells as well as lymphocytes were significantly (p ≤0.05) elevated in MWCNT+ESAT-6-instilled PPARγ KO mice compared to wild-type controls (Table 1) [22]. In response to MWCNT+ESAT-6 instillation, BAL cells from both wild-type and PPARγ KO mice displayed significantly (p ≤0.05) elevated gene expression of the Th1 transcription factors T-Bet and STAT4 as well as the Th1 cytokine, IFN-γ, compared to vehicle controls (Figure 5A). In contrast, levels of Th2 markers, IL-4 and GATA3, did not significantly differ from those of vehicle controls in either wild-type or PPARγ KO mice (Figure 5B). Interestingly, MWCNT+ESAT-6 significantly (p ≤0.05) elevated expression of the Th17 transcription factor RORγt. in both PPARγ KO and wild-type BAL cells compared to vehicle controls (Figure 5C). In PPARγ KO mice, IL-17A expression levels (Figure 5C) were also significantly (p ≤0.05) elevated by MWCNT+ESAT-6 compared to vehicle control. Overall, levels of Th17 markers were significantly (p≤0.05) elevated in MWCNT+ESAT-6-instilled PPARγ KO mice compared to wild-type (Figure 5C).

**Figure 6:**
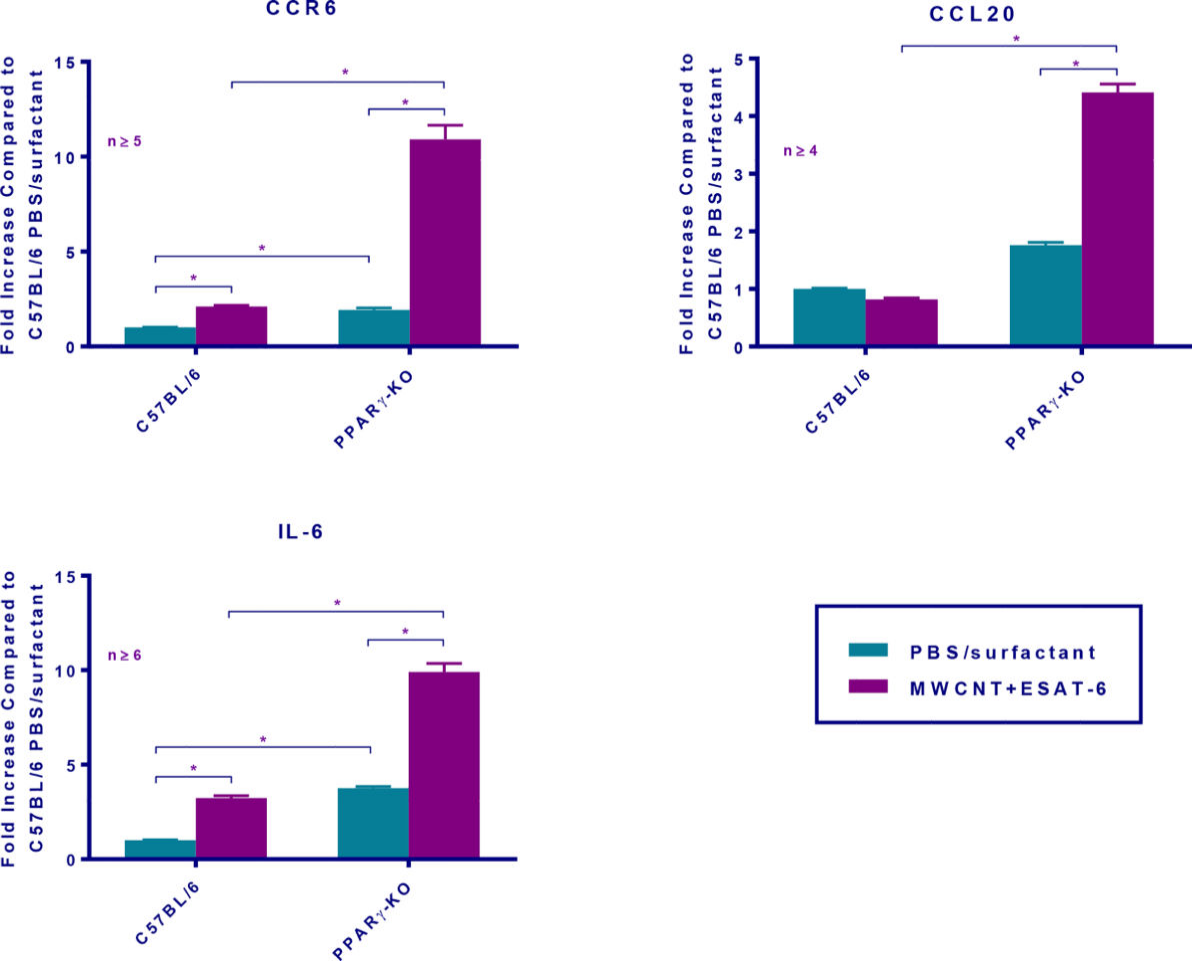
MWCNT + ESAT-6 instillation increases Th-17 development and recruitment markers in BAL cells from C57Bl/6 and PPARγ KO mice. qPCR analyses for CCR6, CCL20 and IL-6 were carried out on BAL cells from C57BL/6 and PPARγ KO mice at 60 days post-instillation with PBS/surfactant and MWCNT + ESAT-6. Data are represented by mean relative fold change ± SEM.; * indicates p 0.05.

Because of the increase in RORγt and IL17A expression in MWCNT+ESAT mice, we hypothesized that CCR6, CCL20 and IL-6 would also be elevated [24,25]. CCR6 is a receptor which works in conjunction with the chemokine CCL20 for recruiting Th17 cells [26]. IL-6 is a cytokine that in association with TGFbeta promotes Th17 cells [27,28]. Our previous studies detected TGFbeta protein in BAL fluid of MWCNT+ESAT instilled PPARγ KO mice [19]. In the present study, CCR6 was markedly elevated as were both CCL20 and IL-6 in PPARγ KO mice instilled with MWCNT+ESAT (Figure 6).

## DISCUSSION

Numerous studies have evaluated immune responses to mycobacterial proteins in murine models for vaccine purposes [29–31]. Immune cell activation has been noted with some types of carbon nanotubes (30). Our previous studies also noted elevated production of IFN-γ and other cytokines in BAL cells in response to MWCNT [18,19]. Here we examined the possibility that changes in T cell activation might occur in response to oropharyngeal instillation of a mycobacterial peptide in association with a carbon nanotube-induced granulomatous condition described in our previous reports [17,18].

The current studies indicate intrinsic elevation of lymph node CD4 T cells in PPARγ KO mice compared to wild-type as has been noted elsewhere (reviewed in Choi & Bothwell) [32]. These results are in line with our previous report indicating the presence of an endogenous Th1 cell inflammatory profile (including elevated IFN-γ) in untreated PPARγ KO mice compared to wild-type [21]. Transduction with a PPARγ lentivirus construct in that study significantly reduced inflammatory mediators [21]. Moreover, PPARγ antagonist treatment of wild-type mice elevated T cell mediator production [21] suggesting that persistent PPARγ deficiency such as noted in sarcoidosis alveolar macrophages [20] may help to maintain an elevated Th1 cell profile.

While lymphadenopathy was significant in mice instilled with MWCNT+ESAT-6 compared to control-treated animals, no clear T effector subset could be identified by qPCR analysis of lymph node cells. These data contrasted with results from BAL cells, where increases in both Th1 and Th17 cell markers correlated with enhanced pathology. In addition, neither wild-type nor PPARγ KO mice showed evidence of granulomatous structures or fibrosis in mediastinal lymph nodes, unlike lung tissues. Lastly, we found no evidence of ESAT-6 in lymph nodes from either PPARγ KO or wild-type mice [19]. Therefore, at 60 days post-instillation much of the activated T cell phenotypes appear to associate with lung tissue rather than with draining lymph nodes.

Relative to ESAT-6 clearance in the lung, our previous report observed a striking difference between wild-type and PPARγ KO mice with respect to pulmonary retention of peptide [19]. Mass spectroscopic scans showed that ESAT-6 was still detectable in lungs of PPARγ KO mice instilled 60 days previously with ESAT-6 alone or in combination with MWCNT. Interestingly, no ESAT-6 signal was detected in similarly instilled 60-day lung tissues from wild-type mice [19]. These findings suggest that lack of PPARγ may impair the efficiency by which foreign peptides are cleared in the lung, allowing more prolonged exposure to local immune cells. Consistent with this idea, PPARγ markedly enhances phagocytosis in alveolar macrophages (reviewed in Croasdell) [33] which intrinsically express high levels of PPARγ in healthy controls compared to patients with sarcoidosis [20].

Our previous studies indicated that combined MWCNT/ESAT-6 instillation of PPARγ KO mice enhanced CD3+ T cell infiltration of lung tissue [19]. Findings from the present BAL cell studies suggest that there is also evidence of elevated Th1 (T-Bet, IFN-γ) and Th17 (RORγt, IL-17A, CCR6) cells within the lungs of wild-type and PPARγ KO mice. Notably, IL-17A-secreting T cells infiltrate lung tissues of mice responding to *Mycobacterium bovis* Bacille Calmette-Guerin infection [34]. In sarcoidosis patients, Th17 cells producing IFN-γ are present in both blood and BAL samples [16]. Interestingly, increased frequency of Th17 cells, unlike that of Th1 cells, was reported to exacerbate radiation-induced fibrosis in mice [15]. Moreover, IL-17 was suggested to play a role in chronic obstructive pulmonary disease (COPD) pathology, although pathways remain to be identified [35].

In our murine model, future studies of lung tissues and nodal lymphocytes are needed to confirm T cell-associated findings since cytokine studies of BAL cells may be confounded by alveolar macrophage production of cytokines such as IFN-γ, IL-4, or IL-17 [36–38]. Moreover, expression of T-Bet has also been noted in macrophages [39]. Future MWCNT studies may benefit from utilizing ESAT-6 specific TCR transgenic mice [29] to more precisely trace T cell and macrophage involvement in pulmonary immunopathology.

Additional studies are also needed to address the question of what regulatory pathways may be operative over time within lung and lymph nodes of PPARγ KO mice to induce Th1 or Th17 differentiation after MWCNT/ESAT-6 instillation. In summary, our current and previous findings suggest that macrophage PPARγ deficiency may create a favorable environment for establishment of immune-associated pulmonary granulomatous disease via pathways that promote: (1) retention of foreign materials in the lung; (2) persistent elevation of T helper lymphocytes; (3) activation of Th17 cells; and (4) exacerbation of histopathologic changes.

## Figures and Tables

**Table 1:** Total Cell Count and Frequency of Inflammatory Subsets in 60 Day BAL.

	Cell Count (x10^5^)	AM (%)	Lym (%)	PMN (%)	Eos (%)
C57BL/6	Mean ± SEM				
PBS/surfactant (n=11)	6.0 ± 0.6	99.0 ± 0.4	1.0 ± 0.4	0.0 ± 0.0	0.0 ± 0.0
MWCNT+ESAT-6 (n=6)	9.9 ± 1.0	94.7 ± 0.4	3.5 ± 0.9	1.3 ± 0.7	0.5 ± 0.3
PPARγ-KO					
PBS/surfactant (n=6)	10.1 ± 1.9	93.2 ± 2.7	4.8 ± 1.3	1.8 ± 1.3	0.0 ± 0.0
MWCNT+ESAT-6 (n=6)	17.3 ± 5.8*	86.8 ± 2.2*	9.3 ± 1.7	3.8 ± 1.5	0.0 ± 0.0

Definition of Abbreviations: AM=Alveolar Macrophage, Lym=Lymphocyte, PMN=Polymorphonuclear Cell, Eos=Eosinophil.

* p≤ 0.05 vs. C57Bl/6 PBS/Surfactant.
